# The mangled extremity and attempt for limb salvage

**DOI:** 10.1186/1749-799X-4-4

**Published:** 2009-02-13

**Authors:** Anastasios V Korompilias, Alexandros E Beris, Marios G Lykissas, Marios D Vekris, Vasileios A Kontogeorgakos, Panayiotis N Soucacos

**Affiliations:** 1 Department of Orthopaedic Surgery, University of Ioannina School of Medicine, Ioannina, Greece; 2 Department of Orthopaedic Surgery, University of Athens School of Medicine, Athens, Greece

## Abstract

**Background:**

The decision, whether to amputate or reconstruct a mangled extremity remains the subject of extensive debate since multiple factors influence the decision.

**Methods:**

Sixty three patients with high energy extremity trauma and attempts at limb salvage were retrospectively reviewed. We analyzed 10 cases of massive extremity trauma where there was made an attempt to salvage limbs, although there was a controversy between salvage and amputation.

**Results:**

All of the patients except one had major vascular injury and ischemia requiring repair. Three patients died. All of the remaining patients were amputated within 15 days after the salvage procedure, mainly because of extremity sepsis. Seven patients required treatment at the intensive care unit. All patients had at least 2 reconstruction procedures and multiple surgical debridements.

**Conclusion:**

The functional outcome should be considered realistically before a salvage decision making for extremities with indeterminate prognosis.

## Background

Occasionally the surgeon is confronts an extremity that is so mangled that salvage is questionable, and a specific answer is almost impossible. Undoubtedly, amputation of a mangled extremity is an unpleasant and devastating process for the patient and the surgeon. On the other hand, prolonged unsuccessful attempts for salvage are highly morbid, costly, and sometimes lethal [1,2]. The decision between amputation and reconstruction remains a matter of controversy [3,4]. Several factors require consideration, such as the extent and severity of vascular injury, bone and soft tissue destruction, the type and duration of limb ischemia, patient's age and previous health status, and the presence of concomitant organ injuries. Efforts should be directed not just to salvage a limb, but to produce a functional painless extremity with at least protective sensation [5,6].

The purpose of this study is to present the magnitude of this important clinical dilemma since the decision between salvage and amputation is vague, and to determine if the clinician will be able to predict amputation in borderline patients using the standard predictive scoring systems.

## Methods

Over a 9-year period from 1996 to 2005, 63 patients with high energy extremity trauma and attempts at limb salvage were retrospectively reviewed. The Mangled Extremity Syndrome Index (MESI) and the Mangled Extremity Severity Score (MESS) were used for scoring both upper as well as lower extremity injuries [7,8]. Although MESS was not developed for the upper extremity injuries, the authors included MESS scoring for making a comparison with MESI. Fifty three patients (84%) ended the postoperative course without any major complication. From the rest ten cases (16%) of massive extremity trauma which had attempts for limb salvage, seven patients (11%) underwent delayed amputation and three patients (5%) died from complications related directly or indirectly to major surgical procedures that followed (Table 1). In these ten cases, the decision between salvage and amputation was not clear. Both scoring systems provided limited diagnostic benefit. Thus, we had an extensive discussion with the patient and his relatives, in order to point out that any attempt at limb salvage might result to major complications and probably a delayed amputation. In addition, even with salvage severe disability was expected.

**Table 1:** Profile of patients with mangled extremity

**Patient**	**Gender**	**Age (years)**	**Mechanism of Injury**	**Upper/or Lower Extremity**	**Ischemia time**	**MESI Score**	**MESS Score**	**Results**
**1**	F	75	MVA	Distal femur	8 h	15	6	Death – Multiple organ failure 24 h postop
**2**	M	29	Farmyard injury	Proximal tibia	7 h	16	6	Sepsis – Amputation 2 weeks postop
**3**	M	25	MVA	Knee joint	6.5 h	15	6	Death – Pulmonary embolism 5 days postop
**4**	M	47	Industrial	Proximal tibia	7.5 h	16	6	Death – Sepsis 2 weeks postop
**5**	M	11	Farmyard injury	Elbow joint	11 h	20	8	Sepsis – Amputation 7 days postop
**6**	M	25	Industrial	Distal humerus	8.5 h	20	6	Sepsis – Amputation 10 days postop
**7**	M	18	MVA	Ankle	6 h	20	8	Sepsis – Amputation 5 days postop
**8**	M	8	Farmyard injury	Ankle	9 h	21	8	Vein/Artery thrombosis – Amputation 3 days postop
**9**	M	25	MVA	Middle femur	-	20	7	Compartment syndrome – Amputation 11 days postop
**10**	M	17	Farmyard injury	Proximal humerus	7.5 h	20	8	Vein/Artery thrombosis – Amputation 3 days postop

MVA: Motor Vehicle Accident

**Figure 1:**
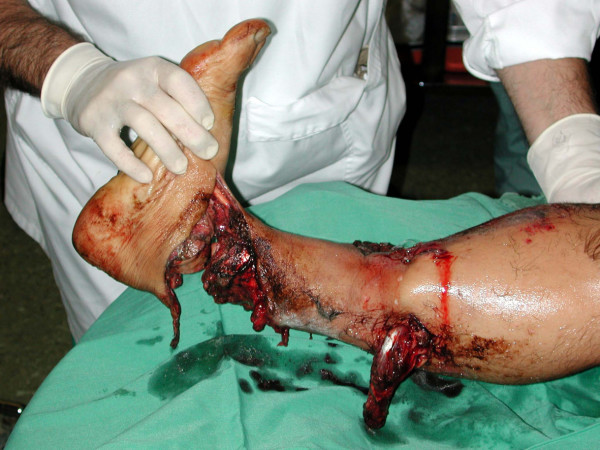
**Eighteen-year-old male patient with severe Gustilo type IIIC injury of the ankle after a motorcycle accident**. An initial attempt for limb salvage with anastomosis of the posterior tibial artery was followed by delayed amputation 5 days postoperatively due to severe sepsis.

Nine patients were males and one female with ages ranging from 8 to 75 years (mean; 27 years). In all cases, the high-energy massive extremity trauma was caused by a combination of crushing and avulsive injury. No guillotine injuries were encountered in this group. The mechanism of injury was labor-related accidents in six patients and motor vehicle accidents in four patients. Gustilo type IIIC fractures (with extensive soft tissue damage and major vascular injury and ischemia requiring repair) were present in nine patients (Figure 1), while one patient had Gustilo type IIIB injury. Seven fractures concerned the lower extremity and three the upper extremity. One patient also had a contralateral transtibial traumatic amputation.

Four patients had concomitant injuries. These included two chest injuries, two head injuries, and one contralateral humeral fracture. None of these injuries was considered as life-threatening. However, in these four patients with a borderline score limp salvage was also attempted.

The time period between the injury and arrival to the operating theater was 6.5 hours. Revascularization was achieved from 6 to 11 hours (mean time; 7.8 hours) (Table 1). Fracture reduction and stabilization was achieved by external fixation in order to decrease the ischemia time, in seven patients with lower extremity injuries, and internal fixation (plate and screws) in three patients with upper extremity injuries. In two patients, temporary arterial shuntings before skeletal fixation was performed.

After bone fixation vascular reconstruction was done using microsurgical techniques. Reconstruction for vascular injury was performed with reverse saphenous vein graft from the contralateral lower limb in eight patients. When both arteries of the extremity were injured, particularly those involving the tibia, both arteries were repaired. No primary repair of the injured nerves was performed.

Fasciotomies were not required because the majority of patients had extensive soft tissue defects. Only one patient with Gustilo type IIIB fracture of the femur underwent delayed fasciotomy.

## Results

In the group of patients who successfully salvaged, the mean MESI and MESS score was 15.5 and 4.8, respectively. However, in the group of patients who underwent secondary amputation or had a fatal outcome both scoring systems varied in identifying a nonviable extremity. In this group, mean MESI and MESS score was 18.3 and 7, respectively.

Although there was no intraoperative death, three patients (5%) died postoperatively. One death was related with massive pulmonary embolism 5 days postoperatively in a 40-year-old female patient with severe Gustilo type IIIC injury of the tibia (with rupture of the popliteal artery above the trifurcation level). A second female patient died within 48 hours of admission. A severe crush injury from motor pedestrian accident resulted in an open Gustilo type IIIC fracture of the right distal femur. The patient underwent surgical repair of both vascular and bony injuries. She also had a contralateral transtibial traumatic amputation. Twenty four hours later she developed myoglobinuria, renal failure, coagulopathy, and multiple organ failure. The third death was due to severe sepsis two weeks after attempting salvage in a patient with Gustilo type IIIC fracture of the tibia.

All the remaining patients (11%) required secondary amputation within 15 days after attempted salvage procedure, because of extensive muscular necrosis (5 patients) and severe extremity sepsis manifested by positive tissue and blood cultures (2 patients). The amputation levels were one above the knee, two below the knee, one Syme's amputation, and three above the elbow amputations. In all cases patient's agreement to this plane of care was obtained after detailed explanation of the new clinical status and all possible alternatives. For these seven borderline patients, amputation was predicted by MESI in 6 patients and by MESS in 5 patients. From the 63 patients, 53 were successfully operated. According to MESI 50 patients were expected to be salvaged and by MESS 47 patients.

Seven patients (11%) required treatment at the intensive care unit from 3 to 41 days. All patients, on the average, were hospitalized for 55 days (range; 30 to 106 days), and had at least 2 reconstruction procedures and multiple surgical debridements. Coverage procedures were done with split-thickness skin grafts in 6 patients and free flap transfer in one patient.

One patient with below knee amputation was able to return to work. One patient with above the knee amputation and one patient with above the elbow amputation were retired on a disability pension. Most patients experience some degree of postraumatic depression and have difficulty to handle the emotional aspects of delayed amputation.

## Discussion

The application of microsurgical techniques has been responsible for significant success in terms of extremity salvage and secondary reconstruction [5,6,9]. However, an attempt for limb salvage should not be made on the basis of what is technically possible [10]. Expertise in, and enthusiasm for, microvascular surgery may lead to costly, highly morbid, and sometimes lethal attempts at preservation of disfunctioned limbs [11,12]. Hansen [13] characterized this approach as triumphs over reason.

Patients who initially confront a threatening injury often focus on the loss of the extremity rather than on the consequences of the limb salvage. Patients undergoing this procedure, will require more complex operations, longer hospitalization, and will suffer more complications than primary amputees. Tornetta and Olson reported on patients who have undergone multiple operations over a period of several years to "heroically" save a leg only to render the patient depressed, divorced, unemployed, and significantly disabled [14]. Unfortunately, "salvage" of a mangled extremity is no guarantee of functionality or employability. It is crucial for the patient and his family to realize that both salvage and early amputation by no means can reassure the patient that will return to a previous normal, pain free extremity [15].

In most of the patients, sepsis and other infection complications may be so severe and persistent that ultimately secondary amputation is required. Bondurant et al. [1] compared primary versus delayed amputations in 43 cases, including 14 primary and 29 delayed ones. Important findings included 6 deaths from sepsis in delayed amputation group compared with none in the early amputation group. The data from our study concur with this data that the delayed amputation was associated with a high risk of extremity sepsis and mortality. It should be clarified that amputation does not necessarily reflect a failure of management but might be the first step to a successful rehabilitation [16].

Although cost should not be a major deciding factor for limb salvage, many patients may be devastated by the cost, not only of medical bills but also of time off work [1]. Fainhurst [17] retrospectively compared the functional outcome of patients who sustained traumatic below knee amputations with that in patients who underwent limb salvage of Gustilo type III open tibial fractures. All patients in the early amputation group returned to work within 6 months of injury, while those who underwent late amputation and salvage returned to work an average of 36 and 18 months after injury, respectively. The authors recommend an early amputation when confronted with borderline salvageable tibial injury. Georgiadis et al. [18] estimated the quality of life by using a questionnaire regarding life satisfaction and disability. Although 35% of the salvage group lost the follow-up, significantly more patients who had had limb salvage considered themselves severely disabled and had more problems with the performance of occupational and recreational activities. On the other hand, most patients dealt with the emotional aspects of amputation in a more positive emotional way of delayed amputation or prolonged and complicated limb salvage [19].

In a recent study, Karladani et al. [20] retrospectively reviewed 18 patients with tibial shaft fractures associated with extensive soft tissue damage. All patients were assessed for their physical function, psychological status, and general function. Almost 90% of the patients were satisfied with the salvage procedure, and if they would be reinjured similarly, 88% of them would prefer limb salvage procedures before amputation. Limitation of the study was, however, the small group size. In contrary, quite a lot of studies have demonstrated that early amputation on the basis of appropriate criteria, improved function and limited the long-term complications [1,2,13].

Several predictive scoring systems have been developed to aid the decision process for limb salvation or amputation. However, almost all classification systems were assessed on retrospective studies, with small number of patients, and patients with known outcomes. In addition, all of the scoring systems are only applied at the time of the initial evaluation, and they do not provide any guiding principles for the decision making in the further treatment course. Another major drawback is that all of the scoring systems apply to specifically for mangled lower extremities, and none of the current classification systems were specifically designed for use in the upper extremity. It is obvious, that a mangled upper extremity has a much greater effect on the patient's life than does a mangled lower extremity. Thus, the criteria for salvage of the upper extremity are quite different from those for salvage of the lower extremity for better salvage functional results and poorer functional prognosis after amputation in the upper extremity. Dirschl and Dahners [11] recommend that mangled upper extremities should be treated on a case-by-case basis and the use of scoring systems should not supplant the surgeon's clinical judgment.

The most widely described scoring systems are: the Mangled Extremity Syndrome Index (MESI) [7], the Predictive Salvage index (PSI) [9], the Mangled Extremity Severity Score (MESS) [8], and the Nerve Injury, Ischemia, Soft-Tissue Injury, Skeletal Injury, Shock, and Age of Patient (NISSSA) Score [21]. Each scoring system has a "cutoff point". If the total score exceeds the critical "cutoff point" primary or early amputation should be considered. However, these scoring systems have been criticized as being too complex and subjective with large variations in interobserver classification of mangled extremity, and as expected none of them is accurate in all cases [22]. Even among experienced surgeons there is disagreement regarding the criteria of these scoring systems, which cannot be used with confidence in clinical practice, because their use has not led to specific outcomes.

Although scoring systems may be helpful, the patient's status cannot simply be summarized by a score number. A closer look reveals that many questions remain unanswered. These systems fail to consider factors related to the patient's quality of life, pain, occupation, age, wishes, social support system, family status, and financial resources. The training and experience of the surgical team may also influence the decision to amputate or reconstruct. Although these considerations are more subjective, undoubtedly they are very important. The true measure of successful limb salvage lies in the overall function and satisfaction of the patient. In our series, the main reason of delayed amputation, despite the initial indication for limb salvage according to MESI and MESS scoring systems, was physician's choice in relation to patient's condition and psychology.

The Lower Extremity Assessment Project (LEAP) is a prospective cohort of patients undergoing limb salvage as compared with those undergoing early amputation [23]. The predictive scoring systems were evaluated to determine whether they were specific, sensitive, and discriminatory in terms of guiding the performance of an early amputation versus limb salvage. Unfortunately, the analysis did not validate the clinical utility of any scales and could not recommend an existing index for determining when to perform amputation versus limb salvage. Injury factors that influence the decision to salvage limbs are muscle injury, absence of sensation, arterial injury, and vein injury. Patient's personal factors played much a less significant role; the most significant of these were alcohol consumption and patient's socioeconomic status [15].

In the present study, both lower and upper extremities injuries were scored using MESI and MESS. The "cutoff point" was 20 and 7, respectively. Among these scoring systems, MESS is the only that derives from a study with a prospective validation trial. The authors used this system because of its simplicity and its ability to score at the time of the initial evaluation without direct observation in the operating room. Although MESS was not designed to score upper extremity injuries, it has been shown that it has 100% specificity and 100% positive predictive value in these injuries [24]. Weak point of this scoring system is its limited sensitivity and negative predictive value when compared to MESI for the upper extremities [24]. In our study, MESI was more accurate than MESS to predict both amputation if amputation was predicted and salvage if salvage was predicted.

## Conclusion

As a majority of cases represent a "gray zone" of unpredictable prognosis, and borderline cases are a dilemma, the decision to amputate or not amputate should not always be made during the initial evaluation. Although scoring systems and "cutoff points" are useful, the final decision for limb salvage should be based on team experience, technical skills, multidisciplinary consultation, tertiary-care facility, and the profile of the patient. Scoring systems should be used only as guides to supplement the surgeon's clinical judgment and experience.

## Consent

Written informed consent was obtained from the patients for publication of their cases and accompanying images. A copy of the written consent is available for review by the Editor-in-Chief of this journal.

## Competing interests

The authors declare that they have no competing interests.

## Authors' contributions

All authors contributed equally to this work. MGL and AVK participated in the design of the study and drafted the manuscript. MDV participated in the design of the study. VAK performed the statistical analysis. AEB and PNS conceived of the study, and participated in its design and coordination and helped to draft the manuscript

Anastasios Korompilias has had the main responsibility for the study and manuscript preparation. All authors read and approved the final manuscript.
